# Effects of rosuvastatin on serum glucose and insulin in hyperuricemic rats

**DOI:** 10.1186/s40360-022-00595-1

**Published:** 2022-09-05

**Authors:** Dilidaer Xilifu, Zumulaiti Tuerxun, Buweiayixiemu Nuermaimaiti, Ayinu Aili, Nijiati Rehemu, Huiping Sun, Xiangyang Zhang

**Affiliations:** 1 Department of CardiologyCardiac care unit, Department of Cardiology, The First Affiliated Hospital of Xinjiang Medical University, 137 Xinyiroad, Xinshi District, ÜrÜmqi, Xinjiang 830011 P.R. China; 2 Respiratory and Critical Care Medicine Center, People’s Hospital of Xinjiang Uygur Autonomous Region, ÜrÜmqi, Xinjiang 830000 P.R. China; 3 Department of Pathology, School of Basic Medicine, Xinjiang Medical University, ÜrÜmqi, Xinjiang 830011 P.R. China

**Keywords:** Rosuvastatin, Hyperuricaemic rat, Fasting blood glucose, Insulin, Glutamic acid decarboxylase antibody

## Abstract

**Background:**

Hyperuricemia is a state in which the serum levels of uric acid (UA) are elevated. This study was to determine the roles of rosuvastatin in fasting blood glucose (FGB) and insulin levels in hyperuricemic rats.

**Methods:**

Thirty-six Sprague-Dawley (SD) rats were randomized divided into the control, model and rosuvastatin groups: the control was given no intervention, the model group was established by administrating yeast extract powder and oxonic acid potassium salt, and the rosuvastatin group was given intravenous administration of rosuvastatin for 28 days in hyperuricemic rats. Serum uric acid (SUA), fasting blood glucose (FBG), fasting blood insulin (FBI), glutamic acid decarboxylase antibody (GADA), oral glucose tolerance test (OGTT) levels, and the ultrastructure of pancreatic β-cells were measured. Also, homeostasis model assessment of insulin resistance (HOMA-IR) scores was computed in three groups.

**Results:**

Compared to the model group, SUA were decreased, while the FBG, GADA, OGTT and HOMA-IR at week 4 were significantly increased in rosuvastatin group. However, FBI was not significantly changed between three groups. It was also showed that the structure of pancreatic β-cells was damaged and the number of β-cells was changed in hyperuricemic rats while they were aggravated in rosuvastatin group.

**Conclusion:**

Rosuvastatin has roles in inducing FGB, GADA, OGTT and pancreatic β-cells damage in hyperuricemic rats.

## Background

Hyperuricaemia is an important risk factor for gout, cardiovascular disease, hypertension, diabetes, and chronic kidney disease [1]. Uric acid (UA) is a diprotic acid of the final decomposition product of purine bases metabolism that can develop into gout [2]. It has been indicated that that elevated serum uric acid (SUA) level is an independent risk factor for the mortality of cardiovascular disease [3]. SUA level is also an independent risk factor for type 2 diabetes mellitus (T2DM) [4, 5]. Elevated SUA levels have been associated with coronary artery disease and also with fasting glucose [6]. Statins are usually used to decrease the serum cholesterol levels and the risk of atherosclerotic cardiovascular diseases [7]. The meta-analysis suggests a significant reduction in SUA levels following statin therapy [8]. Nevertheless, a longitudinal cohort study with large sample size shows that statin can induce the risk of new-onset T2DM [9]. The inhibitory effect of statin on cardiovascular events is greater than the risk of DM [10, 11]. It is unclear why statins have opposing effects on lipids and glucose.

Pharmacological treatment against UA may have therapeutic effects on cardiovascular disease. However, we found that although rosuvastatin reduces SUA levels and improves endothelial function in hyperuricemic rats, fasting blood glucose (FBG) levels in the treated group are still higher than those in the control and model groups after rosuvastatin administration [12]. In this study, we carried out hyperuricemic rat model and treated rats with rosuvastatin calcium to determine the roles of rosuvastatin in FGB and fasting blood insulin (FBI) levels in hyperuricemic rats.

## Methods

### Animal protocol

A total of 36 male Sprague-Dawley (SD) rats (age of 8 weeks, weight of 211.7 ± 21.09 g) were provided by the Experimental Animal Center of Xinjiang Medical University (ÜrÜmqi, Xinjiang, China). The rats were housed individually in specific pathogen-free conditions at a constant temperature (20–22 °C) and humidity (45–55%) with a 12 h light-dark cycle. Rats were fed with a commercial laboratory diet and allowed food and water ad libitum for the duration of the study. After 1 week of an adaptation period, they were randomly divided into three groups: the control (normal rats without any intervention), the model (hyperuricemic rats treated by normal saline) and rosuvastatin (hyperuricemic rats treated by rosuvastatin) groups (*n* = 12 in each group). The hyperuricemic rat model was established by given intravenous administration of yeast extract powder (21 g/kg/day, Beijing Aoboxing Biological Technology, Beijing, China) with standard feed (1:4), and intraperitoneal injection with oteracil (OA) potassium (200 mg/kg/day, Sigma-Aldrich, Munich, Germany) for consecutive 45 days [13]. The hyperuricemic rats were induced by intragastrical administration of rosuvastatin (10.0 mg/kg/day) for 28 days in rosuvastatin group. Rats were weighed twice a week. The study was strictly performed in accordance with the Guide for the Care and Use of Laboratory Animals [14]. The protocol was approved by the Ethical Committee of the First Affiliated Hospital of Xinjiang Medical University (No. 20100310003).

### Specimen collection

The blood sample (2 ml each rat) was collected at 10 am after fasting over night by Retro-orbital bleeding at week 0, 2 and 4. After separation by centrifugation at 2000 rpm for 15 min, plasma was collected for the subsequent analysis.

### Biochemical index detection

After 4 weeks of treatment, SUA, FBG, FBI and GADA in serum were measured. OGTT levels were assayed by oral administration of 40% glucose solution (2 g/kg body weight), and blood samples were collected from the tail vein at 0 and 120 min to measure blood glucose levels (Accu-Chek Performa glucometer, Roche Diagnostics, USA). The insulin levels were measured by enzyme-linked immunosorbent assay (ELISA; EZRMI-13 K, EMD Millipore Corporation, St. Charles, MO, USA). The homeostasis model assessment of insulin resistance (HOMA-IR) scores was computed as follows: HOMA-IR = FBI (μU/mL) × FGB (mg/dL)/22.5) [15].

### Hematoxylin-eosin (HE) staining

After sacrificed by cervical dislocation, the pancreas of rats were collected, weighed, fixed, embedded in paraffin, and sectioned to a 5-μm thickness. Insulin was stained using a DAB Peroxidase Substrate Kit (Fuzhou Maixin Biotech, Fuzhou, China) and counterstained with eosin to observe morphological and quantitative cell changes under a microscope (Olympus, Tokyo, Japan).

### The ultrastructure observation of the rat pancreatic islets

The rest of all rats were perfused with Karnovsky fixative solution: solution A: mixed 2 g paraformaldehyde with 40 ml water at 60 °C on a stir plate, and added 2–6 drops of 1 mol / L NaOH slowly until the solution cleared; solution B: mixed 10 mL 25% glutaraldehyde with 50 ml 0.2md/l sodium dimethylarsenate at pH 7.3. Store the two liquids in 4 L before use, and then mixed them into 100 mL stationary solution.

A peristaltic pressure pump (Watson MHRE200) powered the perfusion. Immediately after cannulation, fixative fluid was introduced. The initial flow rate of the perfusate was low and should be gradually increased over a short period of time after showing the signs for which the fixative solution was effective. The fixation procedure lasted approximately 10 min and required 500 mL of fixative solution per adult rat.

There is no need to monitor the pressure during perfusion. The catheter was made from a 21 g hypodermic needle that is bent at the midpoint and ground flat at the tip. A drop of epoxy resin was placed on one side of the needle tip and then applied around the tip to facilitate the insertion of the catheter into the pancreas, and after insertion, the catheter and pancreas were clamped in place. During manipulation and intubation, the peristaltic pressure pump should be kept at low speed to prevent bubbles from entering the blood vessels. When the intubation was fixed in a proper position, the pancreas was immediately cut while the speed of the pump was accelerated and the fixation solution was introduced to start the fixation. The tissue block or sections should be cut thin enough (no more than 1 mm) to allow for osmium tetroxide penetration. The procedure for osmium tetroxide post fixation, dehydration and embedding was the same as that described above. The perfusion method adopted here was simple and reliable, which could minimize the time between the beginning of anesthesia and effective fixation.

### Statistical analysis

All data presented as the mean ± standard deviation (SD) was analyzed by Statistical Product Service Solutions (SPSS) software version 24 (IBM, Armonk, NY, USA). Statistical analyses were performed by analysis of variance (ANOVA) following Dunnett’s *t*-test for multiple comparisons as appropriate. *P* < 0.05 was considered markedly different.

## Results

### SUA, FBG, FBI, HOMA-IR, OGTT and GADA levels in hyperuricemic rats after administration of rosuvastatin

**Fig. 1:**
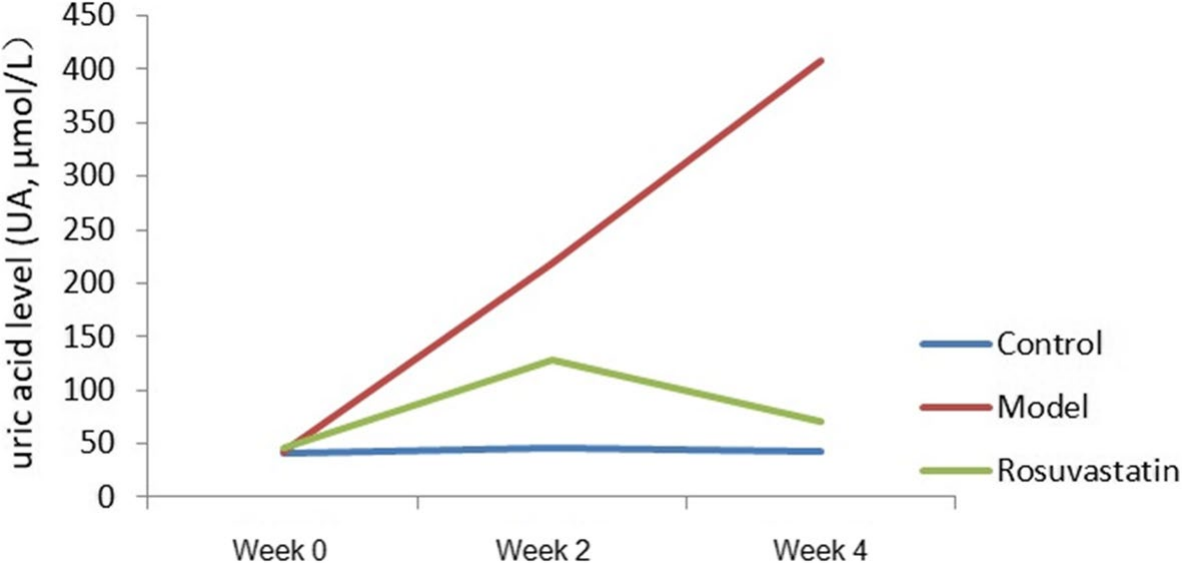
Serum uric acid level changes in the experimental rats at week 0, week 2 and week 4

After administration of rosuvastatin, SUA, FBG, FBI, HOMA-IR, OGTT and GADA levels were detected in control, model and rosuvastatin groups. It was suggested that, compared with the control group at week 2, the SUA and FBG levels increased markedly in the model and rosuvastatin groups (*P* < 0.05). However, there were no notable differences between the rosuvastatin and model group at week 2 (*P* > 0.05). At week 4, the SUA level continued to increase in the model group compared to the control group (*P* < 0.05). SUA was significantly lower in rosuvastatin group than that in the model group (*P* < 0.05), decreased significantly and returned to the level of normal animals (*P* < 0.05; Fig. 1 and Table 1).

**Table 1:** Changes of serum uric acid levels in rats of each group (mean ± SD)

Groups	SUA (μmol/L)		
	Week 0	Week 2	Week 4
Control	41.08 ± 1.39	46.23 ± 3.11^bc^	43.21 ± 2.09^bc^
Model	43.41 ± 5.67	219 ± 17.54^ac^	408.17 ± 4.16^ac^
Rosuvastatin	45.13 ± 0.37	128.75 ± 5.16^ab^	71.00 ± 6.16^ab^

^a^*P* < 0.01, vs. control group; ^b^*P* < 0.01, vs. model group; ^c^*P* < 0.01, vs. rosuvastatin group

**Fig. 2:**
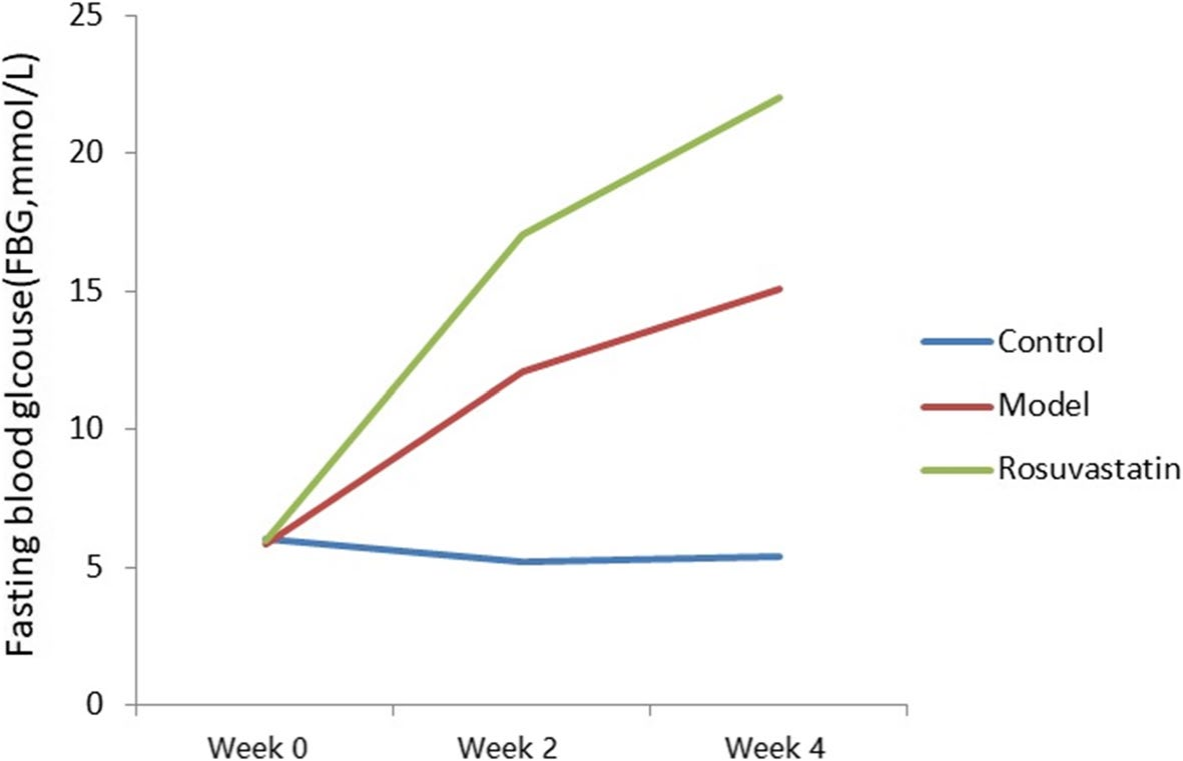
Changes in FBG levels of experimental rats at week 0, week 2 and week 4

**Fig. 3:**
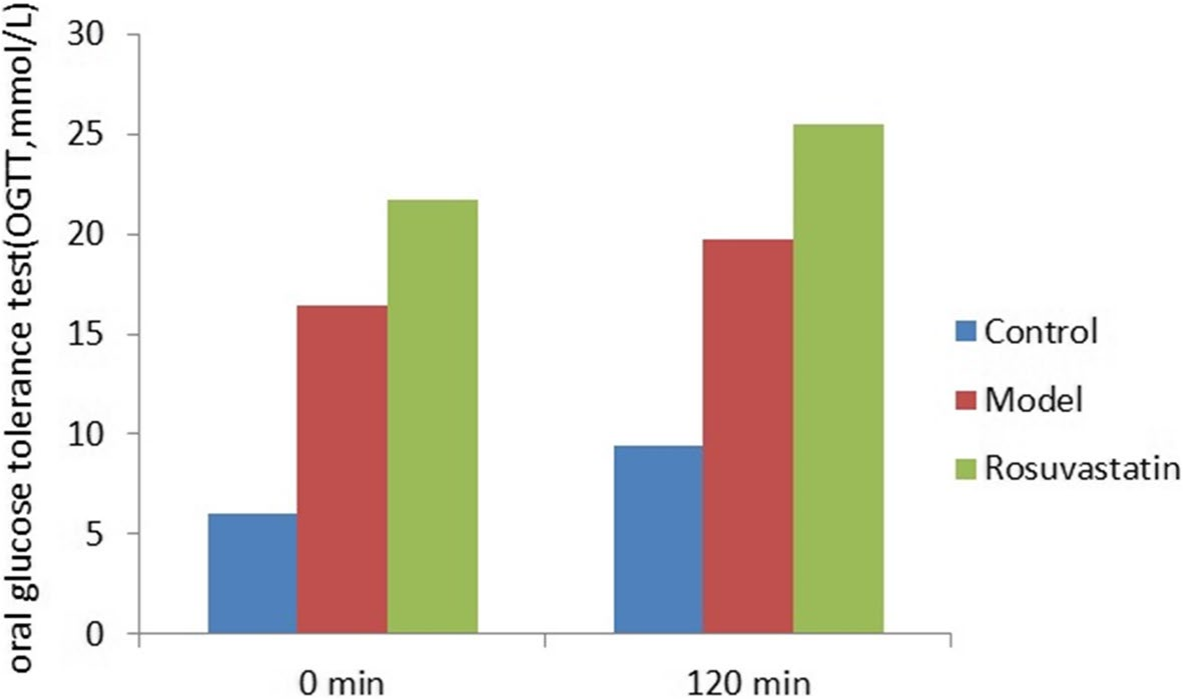
OGTT levels of experimental rats at 0 and 120 min

**Fig. 4:**
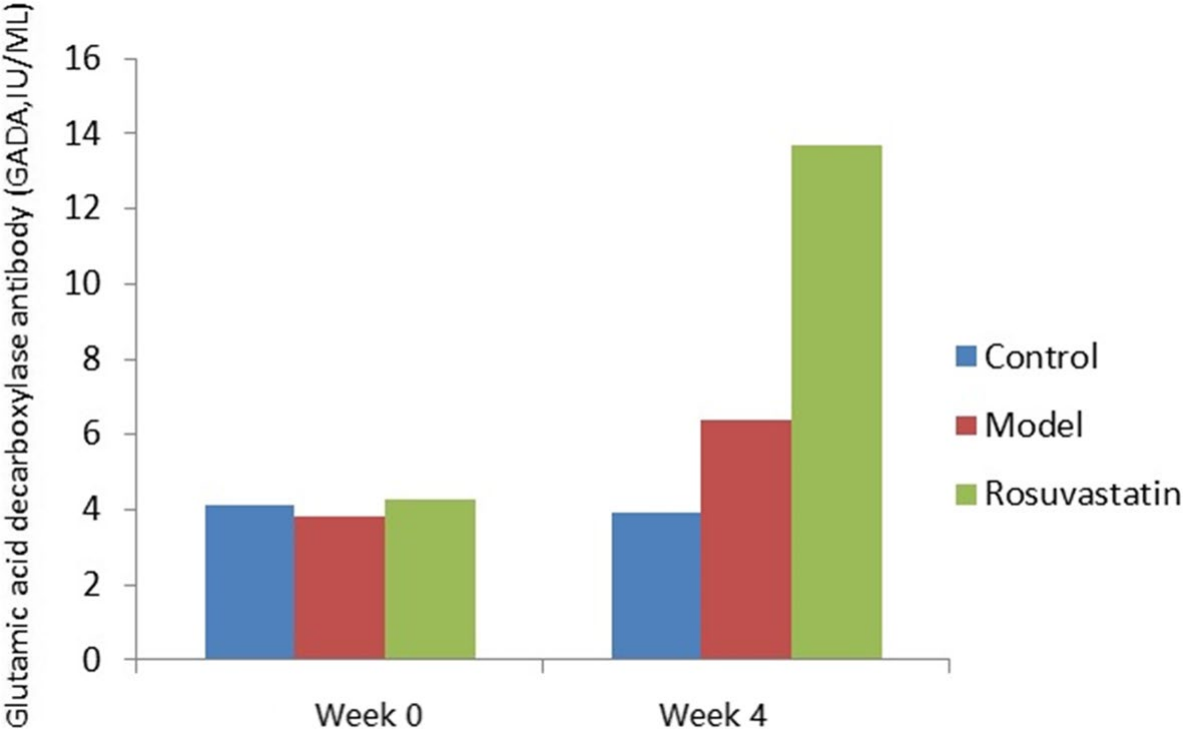
Changes in GADA levels of experimental rats at week 0 and week 4

**Fig. 5:**
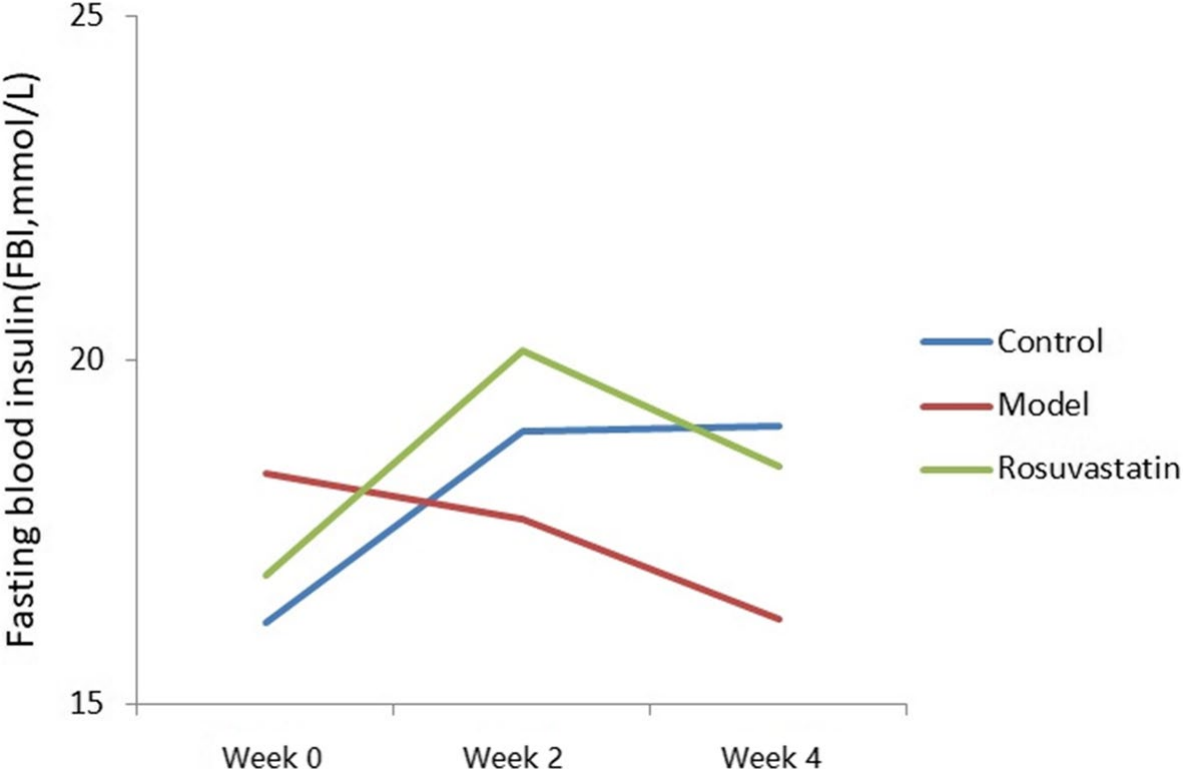
Changes in FBI levels of experimental rats at week 0, week 2 and week 4

At weeks 2 and 4, the FBG levels were significantly higher in the model and rosuvastatin groups than in the control. (*P* < 0.05; Fig. 2 and Table 2). Compared to the model group at week 4, FBG (Fig. 2 and Table 3), OGTT (Fig. 3 and Table 3) and GADA (Fig. 4; Table 4) levels in the rosuvastatin group were significantly increased (*P* < 0.05), but its FBI remained unchanged (Fig. 5 and Table 3).

**Table 2:** Changes of FBG, FBI and MOMA-IR levels in rats of each group (mean ± SD)

Groups	FBG (mmol/L)			FBI (mmol/L)			HOMA-IR		
	Week 0	Week 2	Week 4	Week 0	Week 2	Week 4	Wee 0	Week 2	Week 4
Control	6.05 ± 0.34	5.19 ± 1.01	5.36 ± 1.04	16.19 ± 5.61	18.97 ± 7.10	19.05 ± 8.21	4.35 ± .33	4.37 ± 0.57	4.54 ± 0.38
Model	5.83 ± 0.73	12.05 ± 3.53 ^a^	15.05 ± 1.37 ^ac^	18.35 ± 4.34	17.69 ± 8.15	16.23 ± 9.08	4.75 ± 0.46	9.47 ± 0.39^a^	11.01 ± 0.61 ac
Rosuvastatin	5.99 ± 0.56	17.05 ± 1.09 ^a^	22.05 ± 2.08 ^ab^	16.87 ± 9.26	20.14 ± 4.34	18.45 ± 8.12	4.49 ± 0.12	15.26 ± 0.16 ^ab^	18.08 ± 0.34 ^ab^

^a^*P* < 0.01, vs. control group; ^b^*P* < 0.01, vs. model group; ^c^*P* < 0.01, vs. rosuvastatin group

**Table 3:** Changes of OGTT levels in rats of each group at week 4 (mean ± SD)

Groups	OGTT (mmol/L)	
	0 min	120 min
Control	6.05 ± 0.34 ^bc^	9.4 ± 0.49 ^bc^
Model	16.46 ± 0.72 ^ac^	19.72 ± 1.41 ^ac^
Rosuvastatin	21.73 ± 0.12 ^ab^	25.49 ± 0.33 ^ab^

^a^*P* < 0.01, vs. control group; ^b^*P* < 0.01, vs. model group; ^c^*P* < 0.01, vs. rosuvastatin group

**Table 4:** Changes of GADA levels in rats of each group (mean ± SD)

Groups	GADA (IU/ML)	
	Week 0	Week 4
Control	4.1	3.9 ^c^
Model	3.8	6.4 ^c^
Rosuvastatin	4.3	13.68 ^ab^

^a^*P* < 0.01, vs. control group; ^b^*P* < 0.01, vs. model group; ^c^*P* < 0.01, vs. rosuvastatin group

**Fig. 6:**
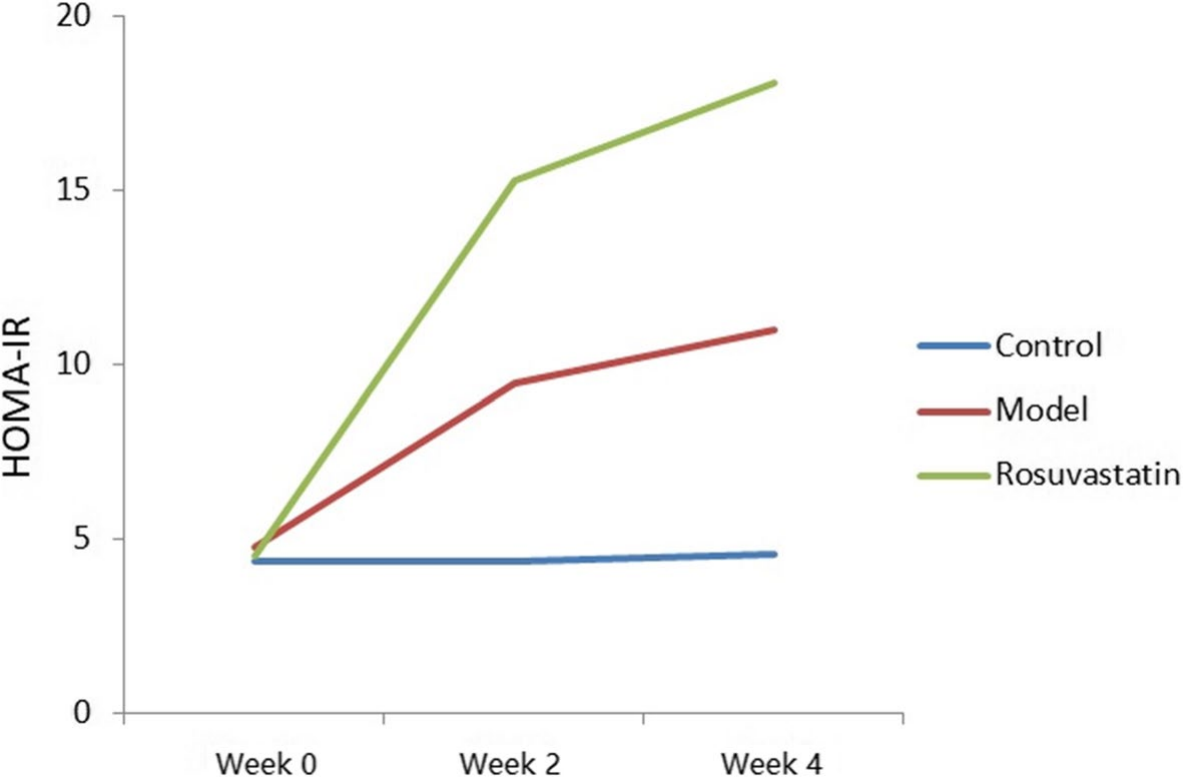
Changes in HOMA-IR levels of experimental rats at week 0, week 2 and week 4

The HOMA-IR values at week 4 were 4.54, 11.01 and 18.08 μU/mL in the control, model and rosuvastatin groups, respectively. There was a significant difference in HOMA-IR between the three groups, and the HOMA-IR values in the rosuvastatin group were shown to be significantly higher than those in the control and model groups (*P* < 0.05; Fig. 6 and Table 2).

### Pathological changes in pancreas of hyperuricemic rats

**Fig. 7:**
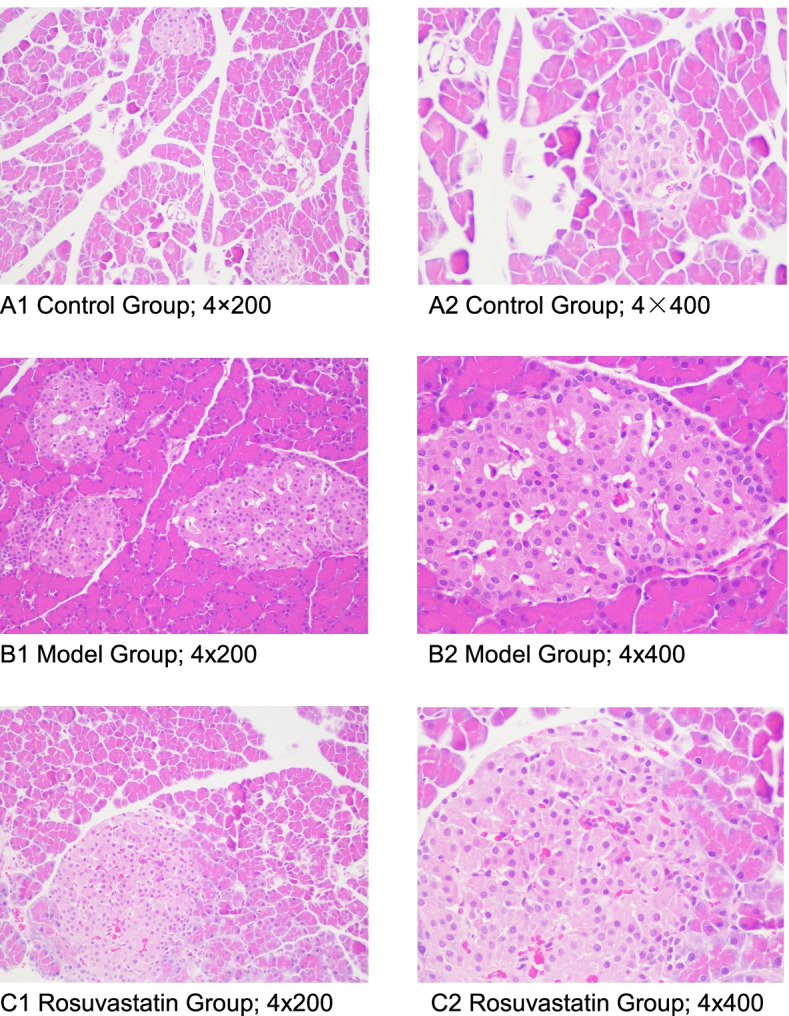
The pathological changes of pancreas islets were observed by HE staining. **A** Control group: A1, magnification of 4 × 200 and A2, magnification of 4 × 400), **B** Model group: B1, magnification of 4 × 200 and B2, magnification of 4 × 400) and **C** Rosuvastatin group: C1, magnification of 4 × 200 and C2, magnification of 4 × 400

Results of HE staining suggested, compared to the control (Fig. 7A), a large number of leukocytes, neutrophils, abscesses, and fibrous tissue proliferation in the pancreas appeared after 2 weeks of treatment in model group (Fig. 7B). At week 4, inflammatory cells infiltrated into the interlobular and intralobular regions in the pancreas of the model group (Fig. 7B). However, these histological alterations were enhanced in the rosuvastatin group, the β-cells structure was disrupted, and most of the islets were enlarged and fibrotic, and some were atrophied compared to the model group (Fig. 7C).

### Ultrastructural changes in pancreatic islets of hyperuricemic rats

**Fig. 8:**
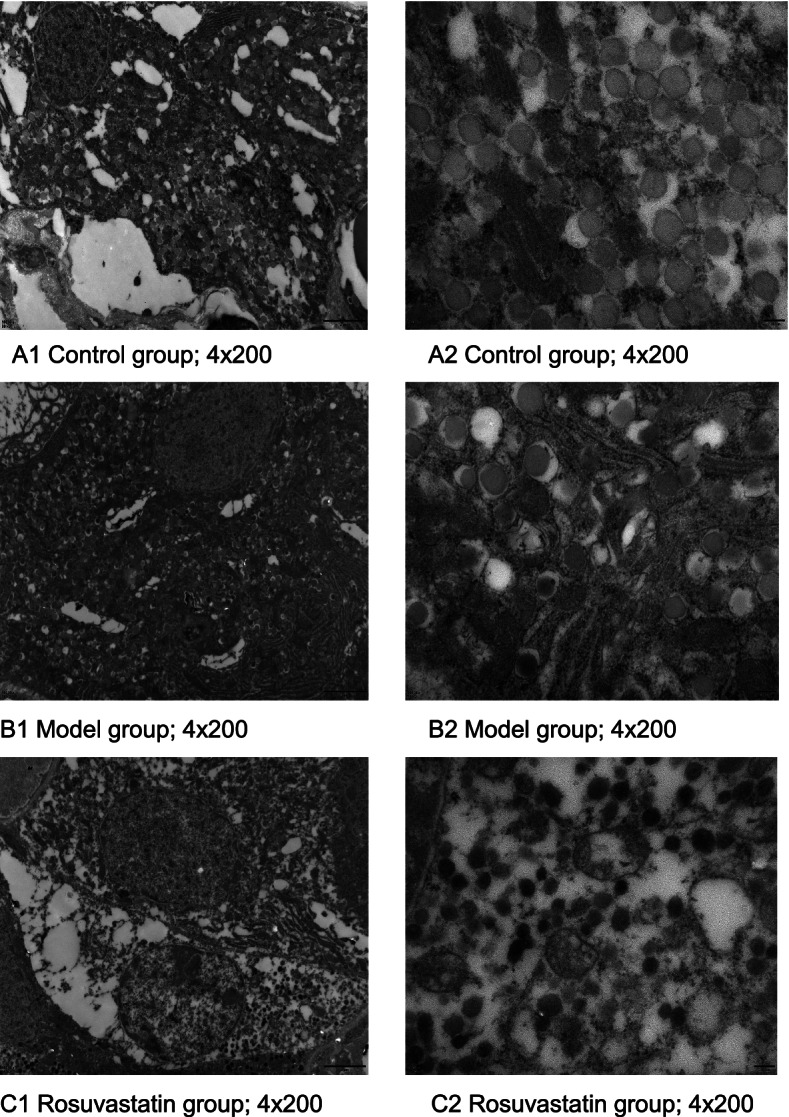
Ultrastructure of pancreas islets. **A** Control group: A1, magnification of 4 × 200 and A2, magnification of 4 × 400), **B** Model group: B1, magnification of 4 × × 200 and B2, magnification of 4 × 400) and **C** Rosuvastatin group: C1, magnification of 4 × 200 and C2, magnification of 4 × 400

Compared with the control group (Fig. 8A), the ultrastructure of islet tissue in hyperuricemic rats showed that the β-cells nuclei in the model group were irregular in shape, some nucleoli were doubled, the morphology and distribution of euchromatin and heterochromatin were basically normal, the β-cells were slightly swollen with more vacuoles and secretory granules, secretory granules differed in size and electron density, surrounding granules were broad, a small number of granules were empty (secretory protein might release), the lumen of the rough endoplasmic reticulum was flattened and slightly dilated, attached ribosomes were segmentally dislodged, Golgi complex was obviously swollen, and the cystic cavity was dilated (Fig. 8A). Compared to the model group, marked swelling of β-cells, lysed amorphous or vacuolated changes in the cellular matrix, and more secretion granules were shown in rosuvastatin group. The secretory granules were small in diameter and high in electron density, with marked edema of mitochondria, flocculent cristae lysis, and vacuolated. Golgi complex was obviously vacuolated (Fig. 8C).

## Discussion

As described in previous studies [16, 17], it was showed that UA is markedly related to the occurrence and development of hyperglycemia. The exact effect of soluble serum urate on glucose metabolism remains elusive. Possible mechanism may be that hyperuricemia induces oxidative stress by increasing reactive oxygen species (ROS) level, which interferes the insulin signaling pathway. It brings out inflammatory state with decreased insulin sensitivity and production, blood glucose uptake and metabolism [18–20]. Additionally, oxidative stress regulates insulin resistance in many cells [21]. Insulin resistance significantly interacts with cardiac oxidative stress [22]. Statins have good clinical benefit in protecting cardiovascular events in hypercholesterolemic patients [23]. However, they have cholesterol-independent pleiotropic effects that influence both insulin and glucose control [24]. A recent clinical study proved that rosuvastatin induces insulin resistance [25]. The results of the present study confirmed previous reports of increased gluconeogenesis caused by statins even with acute treatment of statins in mice [26]. Additionally, a large-scale clinical trial has suggested that rosuvastatin increases the incidence rate of new-onset DM [27, 28]. Our results showed that rosuvastatin markedly decreases the SUA levels while particularly enhances serum FBG, OGTT and GADA levels at week 4.

Rosuvastatin has an effect on glycemic control and systemic insulin resistance [29]. In a recent study, rosuvastatin (10 mg/day) reduces HOMA2 percent-β-cell function and induces HOMA2-IR. Our study revealed a trend towards increased insulin resistance in hyperuricemic rats treated with rosuvastatin [23]. This study showed that rosuvastatin not only enhances the FGB levels, but also worsenes islet damage in hyperuricemic rats. Statins plays a notable role in the pathogenesis of DM, the exact reason remains unclear. Therefore, the possible mechanisms are as follows [30]: First, statins inhibit insulin secretion through calcium channels in pancreatic β-cells. Second, hepatic gluconeogenesis is enhanced, and glucose uptake in surrounding tissues (such as muscle and fat) is disordered. Third, statin treatment decreases the translocation of glucose transporter 4 to plasma membrane. Fourth, statin treatment reduces other pivotal downstream factors, such as coenzyme Q10, farnesyl pyrophosphate, and geranylgeranyl pyrophosphate [30].

It’s important to mention that, in our study, we also found that GADA is obviously enhanced in the rosuvastatin group while the FBI is still unchanged. The possibility of diabetes can be predicted by GADA in risk human [31]. T1DM is mainly caused by autoimmune destruction of pancreatic β-cells. Additionally, in patients with slowly progressive T1DM, the residual β-cell function of low-affinity GADA retains longer [32]. Hence, it is necessary to determine the affinity of disease-specific autoantibodies [33]. The main mechanism may be that the destruction of β-cells also leads to a humoral response with production of antibodies against β-cell autoantigens, with GADA being the most common antibody present in autoimmune diabetes. Glutamic acid decarboxylase-65 (GAD-65) is a major target for autoantibodies in type-1 insulin dependent diabetes mellitus (IDDM), with at least 80% of newly diagnosed patients, or pre-diabetic individuals, possessing these antibodies. Actually, GADA reflects the immunological state in the pancreas of GADA-positive patients with autoimmune diabetes. Recent research indicated thatstatins can inhibit the de novo synthesis of cholesterol and may produce harmful immune inflammatory reactions in pancreatic β-cells. The inhibition of HMG-CoA leads to increased low density lipoprotein (LDL) receptor to enhance the uptake of low density lipoprotein cholesterol (LDL-C), so as to supplement the intracellular reserve. However, the intracellular fate of the two may be different. The oxidation of plasma-derived LDL-C can induce an inflammatory cascade through the immune system, damage pancreatic β cells, and lead to decreased insulin secretion.

Additionally, cytokine-induced nitric oxide (NO) overproduction can induce β-cell apoptosis by activating calpain. In our preliminary study, statins are beneficial to endothelial function by up-regulating the production of NO. Therefore, it is uncertain whether this will negatively regulate β-cells. Furthermore, HDL-C inhibits β-cell apoptosis, while LDL-C induces apoptosis especially after oxidative modification. In general, the interaction between inflammation, oxidation and apoptosis may be triggered by statins, which may be the pathogenesis of DM during long-term statin treatment. This could explain the observed increase in high blood glucose levels with rosuvastatin treatment in hyperuricemic rats.

There are some limitations in this study. First, the changed serum cholesterol levels remain unknown. Hence, the cholesterol produce conditions of the experiment are still questionable. Second, we only used one kind of statins in this study. Whether other statins have similar effects needs a further study.

## Conclusion

We found that rosuvastatin generated or worsed a new onset diabetes in hyperuricemic rats which might be linked to modulation of SUA, FGB, GADA, OGTT and pancreatic β-cells. Elucidation of the mechanisms underlying the development of diabetes in association with statin use may help identify novel preventative or therapeutic approaches to address this problem and/or help design a new generation statin without such side effects.

## Data Availability

All data generated or analyzed during this study are included in this published article.
